# Stem Cells, Inflammation, and Fibrosis

**DOI:** 10.1155/2016/3891386

**Published:** 2016-02-24

**Authors:** Vladislav Volarevic, Majlinda Lako, Miodrag Stojkovic

**Affiliations:** ^1^Centre for Molecular Medicine and Stem Cell Research, Faculty of Medical Science, University of Kragujevac, 69 Svetozara Markovica Street, 34000 Kragujevac, Serbia; ^2^Institute of Anatomy, University of Bern, Baltzerstrasse 2, 3000 Bern 9, Switzerland; ^3^Institute of Genetic Medicine, Newcastle University, Central Parkway, Newcastle upon Tyne NE1 3BZ, UK; ^4^Spebo Medical, 16 Norvezanska Street, 16000 Leskovac, Serbia

Most organs are susceptible to fibrotic diseases and currently fibroproliferative diseases are believed to be responsible for around 45% of deaths in developed countries. Although considerable efforts are being devoted to the search for antifibrotic treatments, there are currently few effective therapies for fibrotic diseases that do not result in severe secondary effects.

Last decades' preclinical and clinical trials have led to application of stem cells as a novel therapeutic agent for the treatment of fibroproliferative diseases.

In this special issue of, several research groups contribute with original research articles as well as review articles that provide novel insights about the possible role of stem cells in modulation of inflammation and fibrosis.

Extensive research has been done on hypertrophic scar and keloid formation that has resulted in the plethora of treatment and prevention methods practiced today. In this issue, B. F. Seo and S.-N. Jung extensively review this body of research examining the mechanism and potential of stem cell therapy in the treatment of excessive scars.

Due to their immunomodulatory ability and capacity for self-renewal and differentiation into tissues of mesodermal origin, mesenchymal stem cells (MSCs) have been proposed as possible new therapeutic agents for the treatment of immune-mediated diseases. MSCs produce cytokines, chemokines, and growth factors that robustly regulate cell behavior in a paracrine fashion during the remodeling process. Due to their immunomodulatory effects and their ability to act on profibrotic factors such as oxidative stress, hypoxia, and the transforming growth factor-*β*1 (TGF-*β*1) pathway, MSC has already been highlighted in preclinical and clinical studies of inflammatory diseases. Accordingly, MSCs as well as their secretomes have been investigated as a novel therapeutic approach for the treatment of inflammatory and fibrotic diseases in several papers published in this issue.

Currently, the most effective therapy for advanced cirrhosis is liver transplantation, but its use is limited because of organ donor shortage, financial considerations, and the requirement for lifelong immunosuppression. An alternative approach such as stem cell transplantation has been suggested as an effective alternate therapy for liver cirrhosis. It is well known that both rodent and human stem cells, in the presence of growth factors, cytokines, chemical compounds, hepatocytes, or nonparenchymal liver cells, are able to differentiate into hepatic progenitor cells (HPC) or hepatocytes* in vitro*, but it is still controversial whether stem cell transplantation can completely regenerate injured liver* in vivo.* Here, A. T. Yang and colleagues report that TGF-*β*1 displays time-dependent dual effect on interplay between HPC and hepatic stellate cells* in vitro* and in animal model of liver fibrosis. Differential stimulation of HPC by TGF-*β*1 for 12 h versus 48 h produces opposing anti- and profibrotic effects indicating the importance of microenvironment and particularly TGF-*β*1 for HPC and hepatic stellate cells crosstalk in the pathogenesis of liver fibrosis.

In line with these findings, L. Zhang and colleagues also highlight the importance of microenvironment and signaling pathways for immunomodulatory capabilities of MSC. They show that Delta-1 overexpression is able to significantly change immunomodulation of MSCs to immune cells, showing potent inhibition of mature T cell proliferation and a slight delay in mature B cell growth, thus providing a potentially novel approach for MSC-mediated modulation of lymphocyte lineage differentiation and development.

Recently published data suggest that MSCs are not constitutively immunosuppressive; they require a “licensing” step provided by molecules of acute phase inflammation, like IFN-*γ* and TNF-*α*. MSCs are immunosuppressive only when exposed to sufficiently high levels of these proinflammatory cytokines while, in the presence of low levels of TNF-*α* and IFN-*γ*, MSCs may adopt a proinflammatory phenotype (MSC1) and enhance inflammation by secreting chemokines that recruit inflammatory cells (particularly Th1, Th17 cells, monocytes, and neutrophils) to the sites of inflammation. The switch toward proinflammatory (MSC1) phenotype or anti-inflammatory (MSC2) phenotype may also depend on MSC stimulation through toll-like receptors (TLRs) expressed on their surface. Polarization to proinflammatory (MSC1) phenotype, important for early injury responses, can be influenced by lipopolysaccharide- (LPS-) dependent activation of TLR4, while double stranded RNA- (dsRNA-) dependent activation of TLR3 may induce the polarization into anti-inflammatory type (MSC2). The balance between these opposing pathways may serve to promote host defense on one hand and at the same time create a loop that prevents excessive tissue damage and promotes fibrosis. Galectin 3 (Gal-3) is protein known to affect proliferation, differentiation, activation, migration, and polarization of immune cells, playing an important role in inflammation and fibrosis. Here, B. S. Markovic and colleagues indicate that Davanat, the newly synthesized inhibitor of Gal-3, could be used for improvement of MSC-mediated polarization of macrophages towards immunosuppressive M2 phenotype. They show that pharmacological inhibition of Gal-3 in MSC enhances their capacity to promote alternative activation of peritoneal macrophages* in vitro *and* in vivo*, in an experimental model of colon inflammation.

Soft dental tissues represent an easily accessible source of stem cells that can be used for autogenic or allogenic cell therapy. Dental stem cells are MSCs capable of self-renewal, multilineage differentiation, and immunomodulation. In this issue, S. Yildirim and colleagues compared proliferation rate, differentiation potential, gene expression, and immunomodulatory effects of dental stem cells isolated from human exfoliated deciduous teeth (SHEDs), dental pulp stem cells (DPSCs), and dental follicle stem cells (DFSCs) on peripheral blood mononuclear cells providing new and important information for cell therapy and regenerative dentistry.

*Vladislav Volarevic* *Vladislav Volarevic* *Majlinda Lako* *Majlinda Lako* *Miodrag Stojkovic* *Miodrag Stojkovic*

